# Ferumoxytol-enhanced magnetic resonance imaging methodology and normal values at 1.5 and 3T

**DOI:** 10.1186/s12968-016-0261-2

**Published:** 2016-07-27

**Authors:** Colin G. Stirrat, Shirjel R. Alam, Thomas J. MacGillivray, Calum D. Gray, Rachael Forsythe, Marc R. Dweck, John R. Payne, Sanjay K. Prasad, Mark C. Petrie, Roy S. Gardner, Saeed Mirsadraee, Peter A. Henriksen, David E. Newby, Scott I. K. Semple

**Affiliations:** 1 British Heart Foundation/University Centre for Cardiovascular Science, University of Edinburgh, Edinburgh, UK; 2 Clinical Research Imaging Centre, University of Edinburgh, Edinburgh, UK; 3 Edinburgh Clinical Research Facility, University of Edinburgh, Edinburgh, UK; 4 Department of Cardiology, Golden Jubilee National Hospital, Clydebank, UK; 5 Department of Cardiology, Royal Brompton Hospital, London, UK

**Keywords:** Cardiac, MRI, Inflammation, USPIO

## Abstract

**Background:**

Ultrasmall superparamagnetic particles of iron oxide (USPIO)-enhanced magnetic resonance imaging (MRI) can detect tissue-resident macrophage activity and identify cellular inflammation. Clinical studies using this technique are now emerging. We aimed to report a range of normal R2* values at 1.5 and 3 T in the myocardium and other tissues following ferumoxytol administration, outline the methodology used and suggest solutions to commonly encountered analysis problems.

**Methods:**

Twenty volunteers were recruited: 10 imaged each at 1.5 T and 3 T. T2* and late gadolinium enhanced (LGE) MRI was conducted at baseline with further T2* imaging conducted approximately 24 h after USPIO infusion (ferumoxytol, 4 mg/kg). Regions of interest were selected in the myocardium and compared to other tissues.

**Results:**

Following administration, USPIO was detected by changes in R2* from baseline (1/T2*) at 24 h in myocardium, skeletal muscle, kidney, liver, spleen and blood at 1.5 T, and myocardium, kidney, liver, spleen, blood and bone at 3 T (*p* < 0.05 for all). Myocardial changes in R2* due to USPIO were 26.5 ± 7.3 s-1 at 1.5 T, and 37.2 ± 9.6 s-1 at 3 T (*p* < 0.0001 for both). Tissues showing greatest ferumoxytol enhancement were the reticuloendothelial system: the liver, spleen and bone marrow (216.3 ± 32.6 s-1, 336.3 ± 60.3 s-1, 69.9 ± 79.9 s-1; *p* < 0.0001, *p* < 0.0001, *p* = ns respectively at 1.5 T, and 275.6 ± 69.9 s-1, 463.9 ± 136.7 s-1, 417.9 ± 370.3 s-1; *p* < 0.0001, *p* < 0.0001, *p* < 0.01 respectively at 3 T).

**Conclusion:**

Ferumoxytol-enhanced MRI is feasible at both 1.5 T and 3 T. Careful data selection and dose administration, along with refinements to echo-time acquisition, post-processing and analysis techniques are essential to ensure reliable and robust quantification of tissue enhancement.

**Trial registration:**

ClinicalTrials.gov Identifier - NCT02319278. Registered 03.12.2014.

## Background

Iron oxide nanoparticles are a class of magnetic resonance imaging (MRI) contrast agents that are generating interest as a method of detecting tissue inflammation. Historically, these nanoparticles were initially used for gastrointestinal, reticuloendothelial system and lymph node imaging [1–3], and subsequently in hepatic and cardiac imaging [4–7]. Recently however, it is in their use as an MRI contrast agent for detecting tissue-resident macrophages that clinical applications are now emerging [8–15].

T2* MRI has been successfully used for over a decade in diagnosing and grading severity of iron accumulation in transfusion-dependent thalassaemia major, and has been instrumental in guiding therapy that improves prognosis, and allows serial disease monitoring [16, 17]. T2* MRI in the assessment of iron accumulation is easily quantifiable, well validated, highly reproducible, clinically robust, and is achievable in a single breath hold [18–22].

Ultrasmall superparamagnetic particles of iron oxide (USPIO) consist of an iron oxide core surrounded by a carbohydrate or polymer coating. These particles can extravasate through damaged capillaries, where they are engulfed and concentrated by tissue-resident macrophages [23]. Gradient echo T2*-weighted (T2*W) sequences are highly sensitive to magnetic field inhomogeneities such as susceptibility artifacts due to the presence of iron, including USPIO. Accumulation of USPIOs in macrophages can be quantified and visualized using T2*W MRI [8, 9] and calculation of, and observing the reduction in, T2* relaxation time due to the presence of iron. Thus USPIO-enhanced MRI can detect tissue-resident macrophage activity and identify localized cellular inflammation within tissues.

In this present study we aimed to observe and quantify the distribution ferumoxytol enhancement following intravenous administration at 1.5 and 3 T MRI and establish a range of normal values for healthy myocardium and other tissue. We also aimed to develop our methodology and describe commonly encountered problems in T2* image analysis of USPIO.

## Methods

This was an open-label observational multi-centre cohort study using human volunteers recruited as part of a larger trial, recruiting patients with cardiac inflammation. The study was performed in accordance with the declaration of Helsinki, the approval of the Scotland A research ethics committee, and the written informed consent of all participants.

### Subjects

Participants were aged over 18 years of age. Exclusion criteria were contraindication to MRI or ferumoxytol infusion, any systemic inflammatory comorbidity (eg rheumatoid arthritis), renal failure (estimated glomerular filtration rate <30 mL/min), pregnancy, breastfeeding and women of child-bearing age not ensuring reliable contraception.

### Magnetic resonance imaging

MRI was performed using 3 T and 1.5 T scanners (Magnetom Verio and Avanto respectively, Siemens Healthcare GmbH, Erlangen, Germany), with dedicated cardiac array coils. All images were acquired using electrocardiogram-gated breath-hold imaging. Routine steady state free precession (TrueFISP) sequences were used to acquire long-axis and short-axis images of the heart. Standard cardiac slice widths (6-mm width with 4-mm gap) and 8 echo times (2.1–17.1 ms range) with matrix size of 256 × 115 were acquired in order to generate T2* maps. The in-plane resolution differed as required for larger or smaller subjects; generally, a field of view of 400 × 300 mm was used with an in-plane resolution of 2.6 × 1.6 mm. T2* relaxation maps were generated before and approximately 24 h after administration of USPIO.

Immediately after the baseline T2* and SSFP cine imaging, breath-held inversion enhancement images were acquired following an intravenous administration of gadolinium contrast medium (0.1 and 0.15 mmol/kg at 3 T and 1.5 T respectively; Gadovist, Bayer Plc, Germany). Optimal inversion time (TI) was determined on a slice-by-slice basis using standard late-enhancement TI-scout protocols. The inversion-recovery late-enhancement short-axis slices were acquired using similar slice positions to the myocardial T2* imaging. The T2* acquisitions also included imaging of the liver, spleen and spine to allow quantification of USPIO accumulation within organs of the reticuloendothelial system.

### USPIO

Intravenous infusion of USPIO (ferumoxytol, 4 mg/kg; Rienso®, Takeda Italia, Italy) was performed immediately following the baseline magnetic resonance scan over at least 15-min using a concentration of 2–8 mg/mL, diluted in 0.9 % saline or 5 % dextrose. Hemodynamic monitoring was conducted throughout.

### Study protocol

**Fig. 1:**
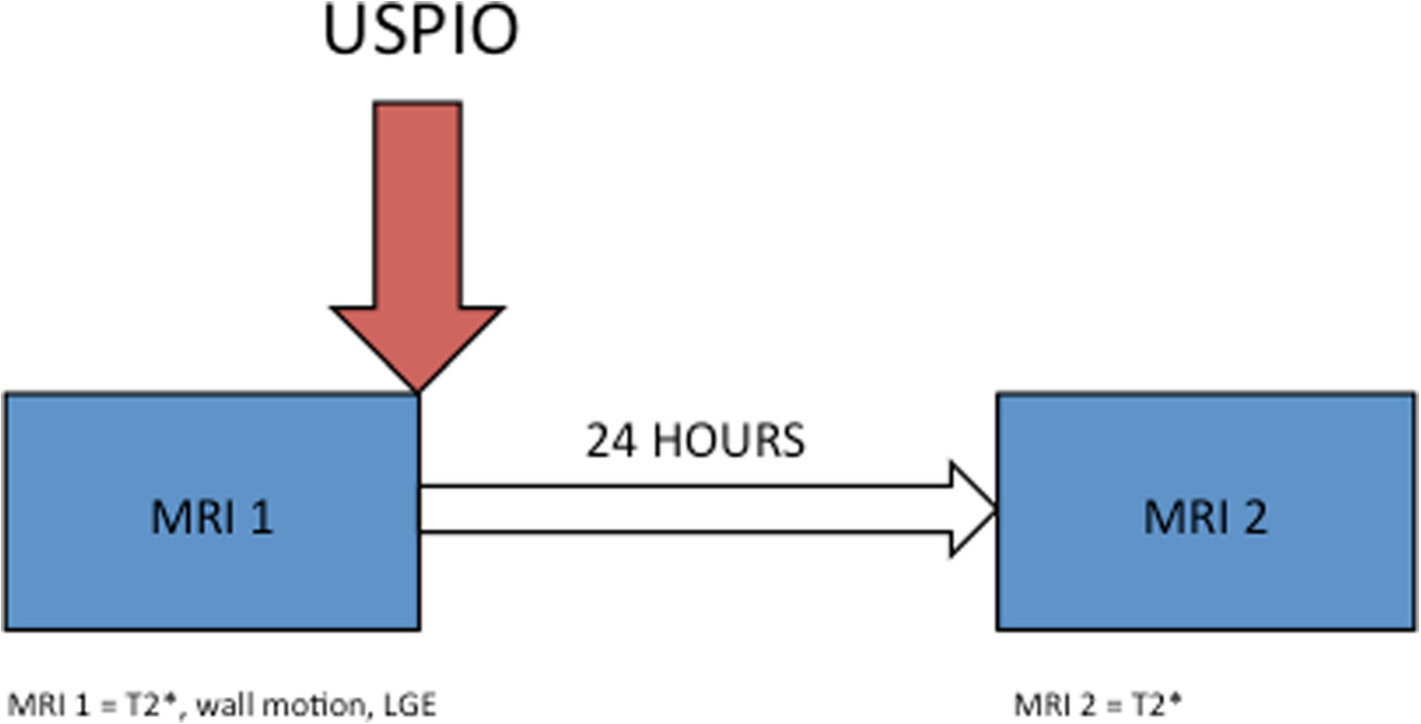
MRI protocol

Volunteers received 2 MRI scans approximately 24 h apart (Fig. 1).

### Image analysis

All T2*-weighted multi-gradient-echo images for each patient were analyzed using Circle CVI software (Circle CVI42, Canada). Regions of interest (ROI) were drawn in the heart using standard cardiac segmentation [24], and panmyocardial values averaged using segments 1–16. Further ROI were drawn in skeletal muscle, kidney, liver, spleen, blood pool (from LV cavity) and bone marrow.

An experimentally determined threshold used in previous work [8] for the coefficient of determination (r^2^ > 0.85) was used to exclude data that did not have an acceptable exponential decay when signal intensity (SI) was plotted against echo time. The inverse of the mean T2* (R2*) for each ROI was then calculated to assess the uptake of USPIO, where the higher the value, the greater the USPIO accumulation.

Late gadolinium enhancement (LGE), ventricular volume and functional analyses were performed using Circle CVI software (Circle CVI42, Calgary, Canada). T2* data were collected immediately prior to USPIO administration. USPIO-enhanced T2* data were collected 24–25 h following ferumoxytol administration.

### Statistical analysis

All statistical analysis was performed with GraphPad Prism, version 6 (GraphPad Software, San Diego, CA). To assess uptake of USPIO in tissues following single administration, R2* increase from pre to 24 h following USPIO were compared using repeated measures one-way ANOVA. Statistical significance was defined as two-sided *p* < 0.05.

## Results

Twenty volunteer patients were recruited in total (10 at 1.5 T, 10 at 3 T). Forty MRI scans and 20 infusions of ferumoxytol were completed over the course of the study. Data from one participant at 1.5 T has been removed due to the presence of LGE, (which was included in the cardiac MR protocol so that we could exclude volunteers with any detectable cardiac MR abnormalities according to standard cardiac MR protocols). All other volunteers that were included had structurally normal hearts. One participant was prescribed antihypertensive medication but had a normal cardiac MR study and was normotensive so the data was retained for analysis. Administration of ferumoxytol was well tolerated with no adverse reactions reported during or immediately after administration in any of the participants.

Participants were predominantly middle aged, with greater numbers of women in both groups (Table 1). There were no differences between 1.5 T and 3 T groups in BMI or ejection fraction at baseline.

**Table 1:** Participant characteristics

	1.5 T	3 T
Number	9	10
Male;Female	3:6	4:6
Age (years)	52 [45.5–61.5]	50 [45.25–53]
Body-mass Index (kg/m^2^)	22.9 [20.1–26.9]	25.9 [22.5–29.4]
Ejection Fraction (%)	63.6 ± 4.9	61.1 ± 4.1

N (%), mean ± SD, or median [interquartile range]

**Fig. 2:**
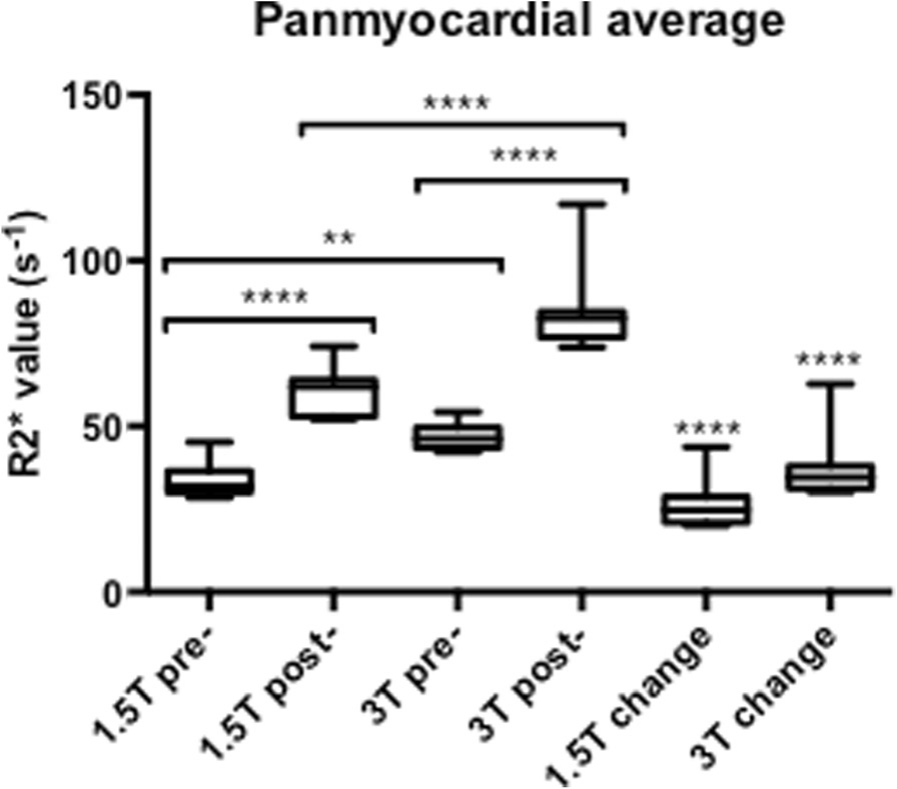
Myocardial R2* pre- and post-USPIO administration at 1.5 and 3 T. Following administration, USPIO was detected by an increase in R2* at 24 h in the myocardium at both 1.5 and 3 T. (**** = *p* < 0.0001, ** = *p* < 0.01)

**Fig. 3:**
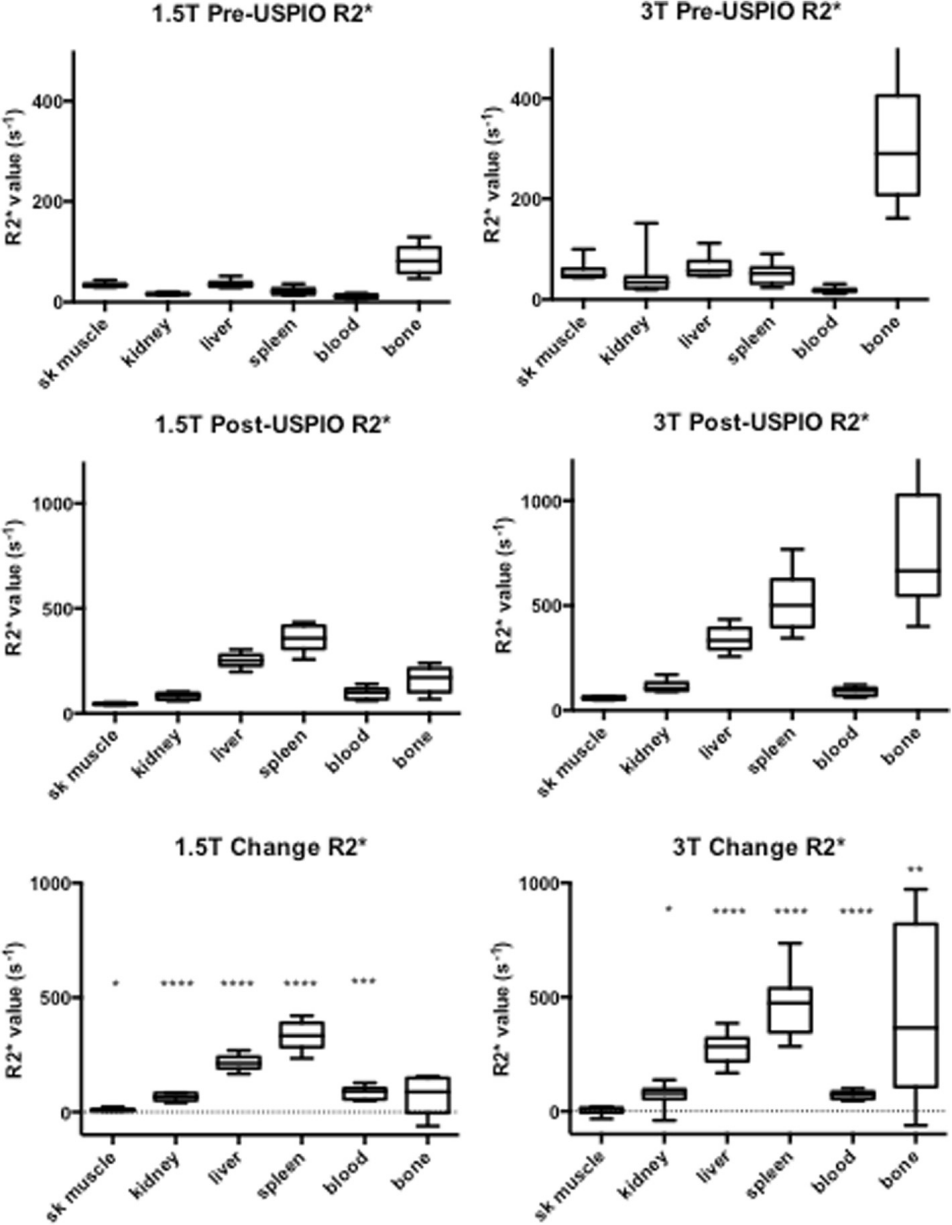
Tissue R2* pre- and post-USPIO administration at 1.5 and 3 T. Following administration, USPIO was detected by an increase in R2*, 24 h after administration in skeletal muscle, kidney, liver, spleen and blood at 1.5 T, and kidney, liver, spleen, blood and bone at 3 T. (**** = *p* < 0.0001, *** = *p* < 0.001, ** = *p* < 0.01, * = *p* < 0.05)

**Fig. 4:**
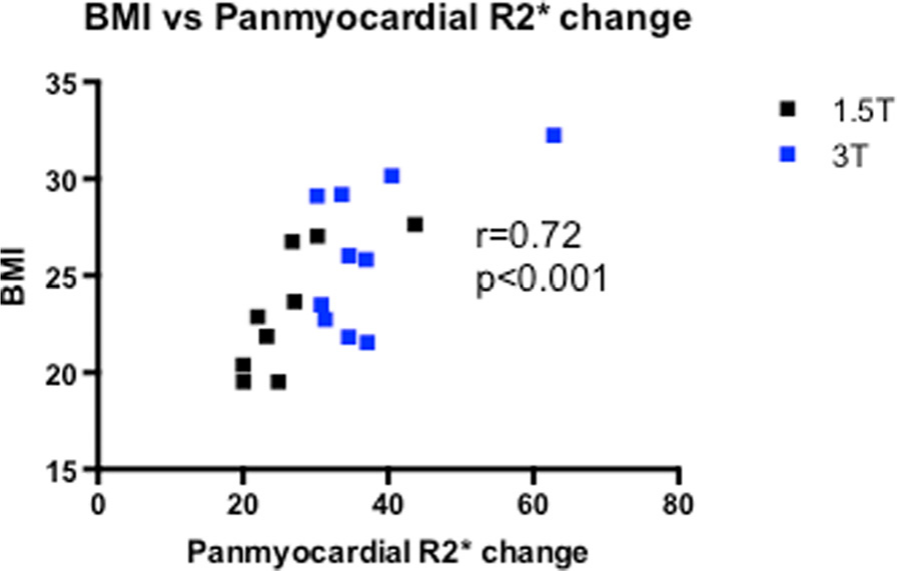
Body-mass Index vs Panmyocardial R2* change at 1.5 and 3 T. Body-mass index correlates with panmyocardial R2* change pre- and post-USPIO

A summary of results is shown in Table 2. At baseline, panmyocardial R2* values were greater at 3 T than 1.5 T (46.9 ± 4.1 versus 33.5 ± 5.4 s^-1^, Fig. 2, *p* < 0.01) as expected. Baseline R2* values were also greater at 3 T in bone (*P* < 0.0001) but no baseline differences were seen between magnetic field strength in all other tissues (Fig. 3, *p* > 0.05 for all). USPIO increased panmyocardial R2* values at 24 h in both 1.5 T and 3 T scanners (*p* < 0.0001 for both). Post-USPIO panmyocardial R2* values were again greater at 3 T than 1.5 T, as expected (84.2 ± 12.4 versus 60.5 ± 7.2 s^-1^, *p* < 0.0001). Panmyocardial change in R2* between baseline and 24 h post USPIO at 1.5 T was 26.5 ± 7.3 s^-1^ and at 3 T was 37.2 ± 9.6 s^-1^ (*p* < 0.0001 for both). Detectable increases in R2* were also observed at 24 h post-USPIO in skeletal muscle, kidney, liver, spleen and blood at 1.5 T, and kidney, liver, spleen, blood and bone at 3 T. (Fig. 3, *p* < 0.05 for all). BMI correlated with the panmyocardial R2* changes due to USPIO contrast (Fig. 4; *r* = 0.72, *p* < 0.001).

**Table 2:** Normal values

	1.5 T Pre-USPIO R2*(s^-1^)	1.5 T Post-USPIO R2*(s^-1^)	1.5 T Change R2*(s^-1^)	3 T Pre-USPIO R2*(s^-1^)	3 T Post-USPIO R2*(s^-1^)	1.5 T Change R2*(s^-1^)
Panmyocardial average	33.5 ± 5.4	60.5 ± 7.2	26.5 ± 7.3	46.9 ± 4.1	84.2 ± 12.4	37.2 ± 9.6
Skeletal muscle	34.7 ± 4.2	44.9 ± 4.7	10.2 ± 5.8	55.5 ± 17.1	59.8 ± 6.6	4.3 ± 16.3
Kidney	16.6 ± 2.0	81.2 ± 15.2	64.6 ± 16.1	43.5 ± 39.1	115.2 ± 28.1	71.8 ± 48.8
Liver	36.0 ± 7.2	252.3 ± 34.3	216.3 ± 32.6	65.3 ± 21.2	340.9 ± 57.8	275.6 ± 69.9
Spleen	22.0 ± 7.7	358.3 ± 59.5	336.3 ± 60.3	51.2 ± 21.1	515.1 ± 137.4	463.9 ± 136.7
Blood	11.3 ± 4.1	96.0 ± 26.6	84.7 ± 27.2	18.8 ± 5.3	91.5 ± 20.9	72.6 ± 18.3
Bone	84.4 ± 29.2	154.3 ± 62.0	69.9 ± 79.9	330 ± 168.7	747.9 ± 277.8	417.9 ± 370.3

Mean ± SD

## Discussion

For the first time, we report a range of normal T2* values in the healthy human heart and other tissues 24 h after ferumoxytol administration at 1.5 and 3 T. We also report problems, solutions and guidance in ferumoxytol-enhanced T2* image analysis.

Following administration, USPIO is detectable by T2* imaging in the myocardium and other tissues at both 1.5 and 3 T. Tissues with small increases in R2* (less than the blood pool) are likely to represent detection of USPIOs within the intravascular space and include skeletal muscle (at 1.5 T only), myocardium and kidney. In contrast, R2* changes that are greater than the blood pool must be due to accumulation of USPIO, either through iron storage, uptake by macrophages or other phagocytes, or sequestered within tissue interstitium. In the absence of tissue biopsies, we cannot be certain, but as the most pronounced R2* changes were seen in the spleen, liver and bone marrow - organs of the reticuloendothelial system - it would appear likely that USPIO is incorporated quickly into tissue-resident phagocytes and macrophages.

Detection of USPIO enhancement in skeletal muscle at 1.5 T but not 3 T is due to generally noisier data seen across all tissues at 3 T. Due to wider data confidence intervals, a larger sample size would be required to detected the same mean change in R2*. The variation in data at 3 T is partly artifact in the images, but also because of the lower values at 3 T (USPIO has a faster T2* decay time at 3 T). With the same sampling echo times, there are fewer data points to construct the decay curve at 3 T than 1.5 T so our error in estimation also increases.

We chose 24 h post USPIO to re-image participants as myocardial signal attenuation at 24 h has shown to be optimal in the myocardium compared to later time points [8, 9]. In view of this, scanning appointments were generally separated by 25 h, and in practice, this regime worked well for both participants and MRI planning. According to previous work [8], we chose a weight-adjusted USPIO dose of 4 mg Fe/kg body weight. However acknowledging that the distribution of USPIO following administration is predominantly in the organs of the reticuloendothelial system and blood pool, this may not be the optimum administration strategy as blood volume does not increase linearly with weight. We found a correlation between BMI and myocardial R2* change, probably due to increased blood pool USPIO concentration in those with higher BMI. We therefore suggest that a fixed dose approach may also be appropriate depending on the application.

**Fig. 5:**
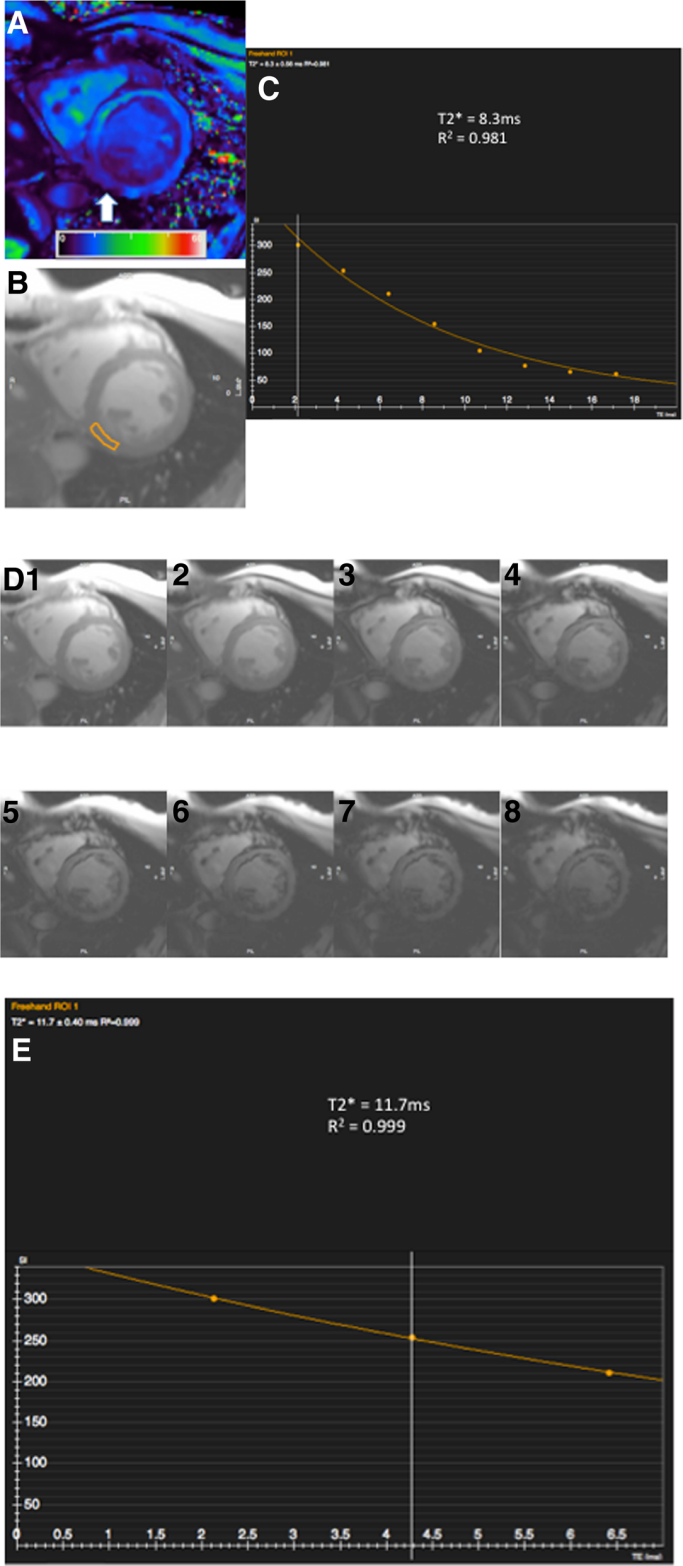
Inferior Blooming artifact. Example illustrating the challenge in assessing whether the inferior myocardial signal attenuation seen arrowed on the T2* colourmap (**a**, scale 0-60 ms) is true or caused by artifact. Drawing a region of interest (**b**) and examining the decay curve (**c**) along with visualising individual echos (**d** *1-8*) helps determine that this is a ‘blooming artifact’ from outside the heart is seen to influence echos 4-8. These can be manually removed, forming a new decay curve (**e**) with improvement in curve fitting (R^2^ value), although with fewer fitting points

Artifacts were commonly encountered with USPIO-enhanced T2* imaging and made data analysis challenging. Post contrast artifacts at the blood-pool to myocardial interface were commonly seen and needed careful exclusion when selecting myocardial ROI. (Fig. 5A) This limited the assessment of USPIO accumulation at the endocardium. Similarly, blooming artifacts from nearby organs with high iron or blood pool USPIO content, such as lung and liver, commonly created signal deficits within the myocardium. In this situation, examination of T2* decay curves and excluding echo times influenced by artifact aided T2* decay curve fitting (Fig. 5).

The advantage of MRI mapping techniques is that visual assessment and objective quantification can be made using the same image, and these are now entering clinical practice. It seems likely that if UPSIO-enhanced MRI is adopted into clinical practice to detect tissue inflammation, T2* mapping would be used for image interpretation. However based on our experiences, we would recommend caution in interpreting maps alone. Signal attenuation seen on the T2* map may be interpreted as tissue USPIO accumulation, but may be due to blooming artifact from nearby susceptibility effects, and close examination of the T2* decay curve, and individual echoes if possible is suggested in order to distinguish accurately between tissue USPIO accumulation and artifact. In theory, setting an r^2^ threshold as we did helps to exclude areas grossly affected by artifact. In practice however, regions with a seemingly acceptable R^2^ may still be influenced by artifact (Fig. 5). Manual exclusion of later echoes (influenced by artifact) from the curve may result in an improvement in R^2^ (a measure of how well the data points fit the curve), however there is the danger that reducing the number of fitting points will in fact reduce the overall sampling accuracy. Clearly, automated software capable of detecting and excluding artifact would be advantageous. This could be achieved by excluding, or applying less weight, to later echo times especially data points at a large distance from the initial decay curve trajectory [25, 26]. It should be noted that like all other MRI sequences, poor data quality heavily influenced by breathing or movement artifact is generally non-interpretable and post processing using automated T2* decay curve fitting software is not likely to provide a remedy.

**Fig. 6:**
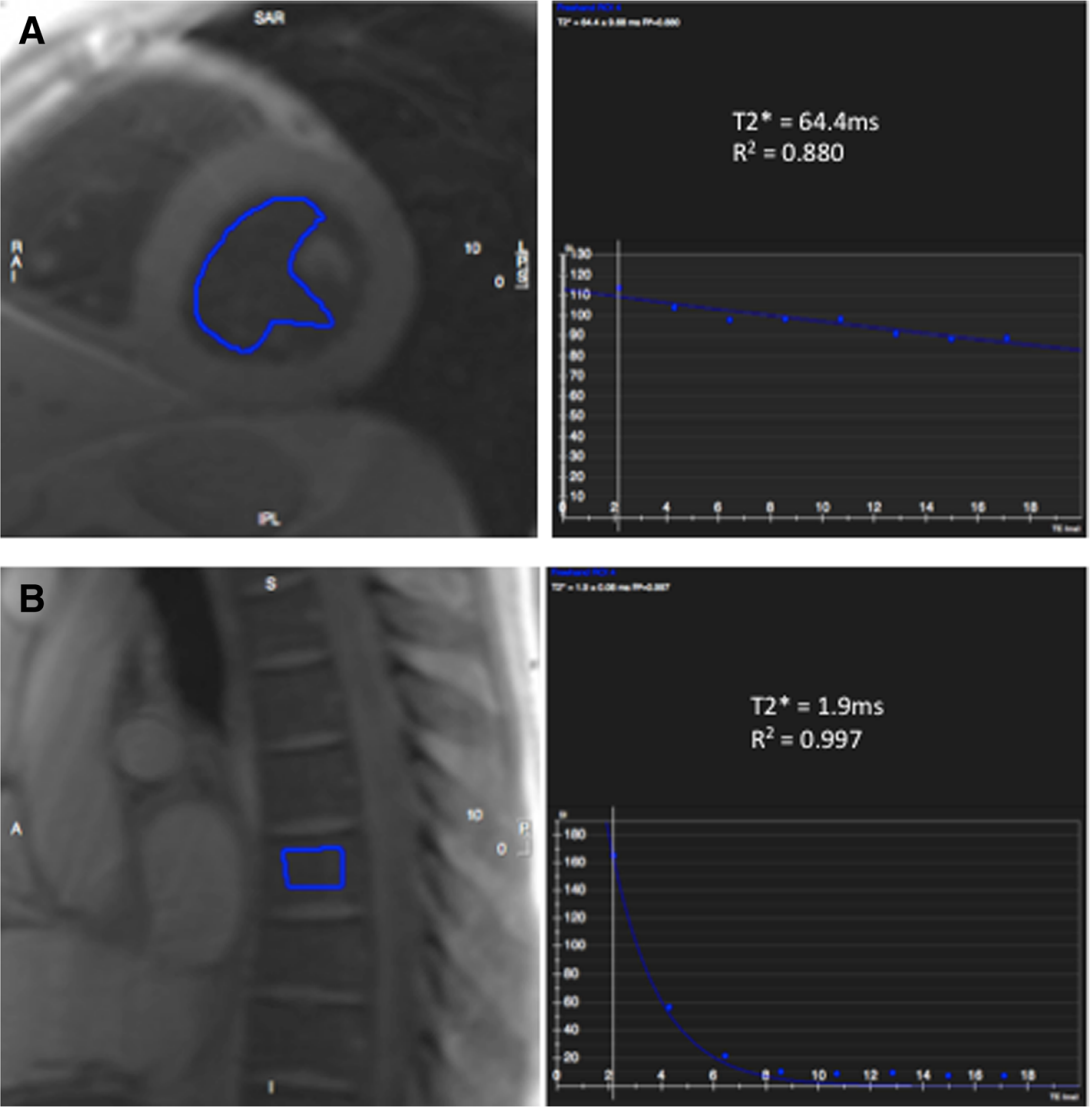
Example of high and low T2* values. Regions of Interest with excessively low or high T2* value (pre-contrast blood pool, **a**, and post USPIO bone marrow, **b**, respectively) can often be difficult to generate an accurate T2* decay curve. Imaging with tissue-spcific echo times will help generate more accurate T2* decay curves

Echo times in this study were specific for cardiac imaging and were selected appropriately. Therefore they were not optimal for imaging tissues with T2* values substantially higher or lower than myocardium. Native blood pool and post USPIO bone marrow (Fig. 6) provide examples of low and high T2* values respectively that we had difficulty accurately fitting a T2* decay curve. With high T2* values, only a short part of the decay curve is plotted over the echo sampling time period, and often the signal has not decayed sufficiently for an accurate decay curve to be plotted. In contrast, regions with particularly short T2* decay times have decayed to a level expected from background noise before sufficient data sampling has been made. Therefore fitting a decay curve from a small number (2–4) of echo times is clearly difficult, and often too much emphasis is placed upon data decayed to the baseline level of background noise in order to generate a decay curve. Allowances can be made for background noise but are of limited value in this instance. We strongly advise applying tissue-specific echo times tailored to the expected T2* value in order to achieve the most accurate decay curves possible.

### Limitations

There are some limitations that should be taken into account when interpreting these data. First, this study has small numbers and a larger cohort of participants should be studied to further validate these normal values. Furthermore, due to geographical reasons, it was not feasible to scan the same participants at both centres so comparison cohorts at 1.5 T and 3 T were different. Despite this, both were healthy volunteers groups and displayed no differences at baseline so we do not feel this has impacted on the results. Finally, due to problems in interpreting high and low T2* values as mentioned above, we recommend caution in interpreting some high non-cardiac R2* values; especially in the organs of the reticuloendothelial system at 3 T. In these organs, the spread of R2* data above the median value appears wide. This is possibly caused by artifact and most evident at 3 T, and may additionally explain why these regions have disproportionally high R2* values.

## Conclusion

We have shown that ferumoxytol-enhanced MRI is feasible at both 1.5 T and 3 T, and suggest a range of expected normal values post-ferumoxytol in a range of tissues. Refinements of dose administration, optimization of acquired echo-times, careful image analysis, and development of post-processing and analysis software capable of excluding common artifacts, are essential to ensure reliable and robust quantification of tissue enhancement.

## Abbreviations

LGE: late gadolinium enhancement
MRI: magnetic resonance imaging
ROI: regions of interest
USPIO: ultrasmall superparamagnetic particles of iron oxide
